# The *Arabidopsis* gene *DIG6* encodes a large 60S subunit nuclear export GTPase 1 that is involved in ribosome biogenesis and affects multiple auxin-regulated development processes

**DOI:** 10.1093/jxb/erv391

**Published:** 2015-08-13

**Authors:** Huayan Zhao, Shiyou Lü, Ruixi Li, Tao Chen, Huoming Zhang, Peng Cui, Feng Ding, Pei Liu, Guangchao Wang, Yiji Xia, Mark P. Running, Liming Xiong

**Affiliations:** ^1^Division of Biological and Environmental Sciences and Engineering, King Abdullah University of Science and Technology (KAUST), Thuwal 23955–6900, Kingdom of Saudi Arabia; ^2^Key Laboratory of Plant Germplasm Enhancement and Specialty Agriculture, Wuhan Botanical Garden, Chinese Academy of Sciences, Wuhan 430074, China; ^3^Institute for Integrative Genome Biology, Center for Plant Cell Biology & Department of Botany and Plant Sciences, University of California, Riverside, CA 92521, USA; ^4^Bioscience Core Lab, King Abdullah University of Science and Technology (KAUST), Thuwal 23955–6900, Kingdom of Saudi Arabia; ^5^Department of Biology, Hong Kong Baptist University, Hong Kong, P. R. China; ^6^Department of Biology, University of Louisville, Louisville, KY 40292, USA

**Keywords:** AtLSG1, *Arabidopsis*, auxin homeostasis, expression pattern, map-based cloning, proteomics, ribosome biogenesis.

## Abstract

The circularly permuted GTPase large subunit GTPase 1 (LSG1) is involved in the maturation step of the 60S ribosome and is essential for cell viability in yeast. Here, an *Arabidopsis* mutant *dig6* (drought inhibited growth of lateral roots) was isolated. The mutant exhibited multiple auxin-related phenotypes, which included reduced lateral root number, altered leaf veins, and shorter roots. Genetic mapping combined with next-generation DNA sequencing identified that the mutation occurred in *AtLSG1-2*. This gene was highly expressed in regions of auxin accumulation. Ribosome profiling revealed that a loss of function of *AtLSG1-2* led to decreased levels of monosomes, further demonstrating its role in ribosome biogenesis. Quantitative proteomics showed that the expression of certain proteins involved in ribosome biogenesis was differentially regulated, indicating that ribosome biogenesis processes were impaired in the mutant. Further investigations showed that an *AtLSG1-2* deficiency caused the alteration of auxin distribution, response, and transport in plants. It is concluded that *AtLSG1-2* is integral to ribosome biogenesis, consequently affecting auxin homeostasis and plant development.

## Highlight

*DIG6* encodes a large 60S subunit GTPase 1 and affects ribosome biogenesis, and auxin response and homeostasis.

## Introduction

Ribosomes are complexes of RNA and protein molecules that are present in the cytoplasm or attached to the surface of the rough endoplasmic reticulum. They are the primary site for protein synthesis and are thus called protein factories. Ribosomes usually consist of one small subunit and one large subunit. Production of these ribosomal subunits is a strictly regulated process. In eukaryotes, assembly begins in the nucleolus, and pre-subunits are exported to the cytoplasm where their assembly is completed. Ribosome biogenesis requires not only the factors involved in processing ribosomal RNA and proteins but also a number of *trans*-acting factors that organize the assembly process. These *trans*-acting factors include GTP- and ATP-binding proteins that are necessary for several energy-consuming steps.

Eukaryotic *LSG1* genes encode a large subunit GTPase (LSG1) that is involved in the maturation of the 60S subunit. This GTPase belongs to a large circularly permuted GTPase family whose members contain a central GTP domain. The structure of this domain is different from the canonical organization of GTPases in that the order of the five motifs is rearranged from G1, G2, G3, G4, and G5, to G4, G5, G1, G2, and G3 (Reynaud *et al.*, 2005). Many members of this GTPase family are associated with ribosome biogenesis. LSG1 is involved in the late maturation steps of pre-60S subunit assembly and participates in the nuclear export of the pre-60S subunit in yeast. However, given that in yeast, LSG1 is a cytoplasmic protein, its regulatory role on the nuclear export of pre-60S subunits is indirect. The effects of LSG1 on the nuclear export of pre-60S subunits are controlled by the nuclear export signal (NES)-bearing protein nonsense-mediated decay 3 (NMD3) (Ho *et al.*, 2000; Gadal *et al.*, 2001; Hedges *et al.*, 2005). NMD3 is a nucleo-cytoplasmic shuttling protein and an adaptor for the export of 60S ribosomal subunits from the nucleus. NMD3 is recruited by LSG1 and RIBOSOMAL PROTEIN L10 (RPL10); the loss of *RPL10* or *LSG1* causes the accumulation of NMD3 in the cytoplasm and ultimately fewer 60S ribosomal subunits (Hedges *et al.*, 2005). In yeast and human cells, loss of function of *LSG1* is lethal (Hedges *et al.* 2005; Reynaud *et al.*, 2005), while in *Drosophila*, the *LSG1* homologue (*NS3*) has been reported to regulate the insulin signalling pathway and control body size (Kaplan *et al.*, 2008). In *Arabidopsis*, there are two orthologues of yeast LSG1—AtLSG1-1 and AtLSG1-2 (Weis *et al.*, 2014)—whose functions in plant development are less studied than are their yeast counterparts. A recent study showed that AtLSG1-2 is involved in the maturation process of 40S proteins such that its loss of function caused multiple phenotypes including small size and incurvature leaves. On the other hand, loss of function of *AtLSG1-1* has little effect on plant development. However, simultaneous loss of both genes is lethal, suggesting that they are required for plant growth (Weis *et al.*, 2014).

Here, the *dig6* (drought inhibited growth of lateral roots) mutant was isolated and characterized. This mutant had reduced lateral root number and strong incurvature in rosette leaves. Map-based cloning identified that the *dig6* mutation occurs in *AtLSG1-2*. The importance of *AtLSG1-2* in ribosome biogenesis was further evidenced *in vivo* by ribosome profiling and proteomics analyses of the mutant. Because the pleotropic phenotypes of *dig6* are reminiscent of auxin-related mutants, auxin distribution, response, and transport were investigated. It was found that auxin response and homeostasis were altered in the mutant. This study highlights the roles of AtLSG1-2 in ribosome biogenesis, and auxin homeostasis and response in plants.

## Materials and methods

### Plant materials and growth conditions

The *Arabidopsis dig6* mutant was isolated for its reduced lateral root growth (Xiong *et al.*, 2006) from an M_2_ population generated by ethyl methane sulphonate (EMS) mutagenized *Arabidopsis* in the Columbia *glabrous 1* (Col-*gl1*) background. Seeds of T-DNA insertion lines and auxin transporter::GFP reporter lines were obtained from the Arabidopsis Biological Resource Center. Seeds were surface sterilized with a 50% bleach solution for 5min, washed with water five times and then planted on half-strength Murashige and Skoog (MS) agar-medium plates. The plates were kept at 4 °C in darkness for 2–4 d and then moved to a growth chamber (CU36-L5, Percival Scientific) at 21 °C under a photoperiod of 16h light and 8h darkness (long-day conditions) or a photoperiod of 8h light and 16h darkness (short-day conditions). For morphological and histological analyses, seedlings were transferred to soil in a growth room under the same growth conditions as in the growth chamber.

### Plasmid construction

To generate the *pAtLSG1-2*::GUS fusion construct, a 2006bp promoter fragment from genomic DNA was amplified by PCR and first cloned into the pENTR^TM^/D-TOPO vector and subsequently cloned into the upstream of GUS in pMDC162 using GATEWAY technology. To construct *35S::AtLSG1-2–YFP* and *35S::YFP–AtLSG1-2*, the full-length *AtLSG1-2* coding region was cloned into the pENTR^TM^/D-TOPO vector and then into vectors pEarlygate 101 or *pEarlygate* 104, respectively, to produce *p35S::AtLSG1-2-YFP* or *p35S::YFP-AtLSG1-2*. Using the same method, *AtLSG1-1* was cloned into the *pEarlygate* 104 vector to construct *p35S::YFP-AtLSG1-1*. Information on primers used in this study is given in Supplementary Table S2 available at *JXB* online.

### Plant transformation

Transgenic plants were generated by transferring plasmids into *Arabidopsis* via the Agrobacterium-mediated flower-dipping method (Clough and Bent, 1998). Protoplasts were prepared from fully expanded healthy leaves of 3–4-week-old plants. *Arabidopsis* protoplast transformation was performed as previously described (Yoo *et al.*, 2007).

### Quantitative real-time PCR

Total RNA was extracted from plant materials using an RNeasy Mini kit (QIAGEN). DNA was eliminated from total RNA using DNase I (NEB) and cleaned with the RNeasy Mini kit (QIAGEN). Cleaned RNA was reverse transcribed using the SuperScript Reverse III reagent (Invitrogen) according to the manufacturer’s instructions. Two micrograms of RNA were used for quantitative real-time PCR (qRT-PCR) analysis. qRT-PCR was performed on a 7900HT Fast real-time PCR system (Applied Biosystems) using SYBR Green PCR Master Mix (Applied Biosystems). Normalization was conducted against the average of housekeeping genes *UBQ10* or *ACT2*. Relative gene expression was calculated by the equation 2^−ΔΔCT^. Three biological replicates were performed.

### Histochemical staining, histological analysis, and microscopy

Plant materials were incubated in ice-cold 80% acetone for 30min, rinsed with 100mM sodium phosphate buffer twice and then incubated in a 1mg/ml of 5-bromo-4-chloro-3-indolyl-β-d-glucuronic acid (x-gluc) solution at 37 °C for a period ranging from 1h to overnight. Stained materials were cleared overnight in 90% ethanol. Samples were examined and photographed under a microscope. For histological analysis, fully expanded fifth leaves were fixed in 2.5% glutaraldehyde with 100mM phosphate buffer (pH 7.2) followed by osmication with 2% osmium tetroxide in 100mM phosphate buffer (pH 7.2). After dehydration with an ethanol series, samples were infiltrated and embedded in Spurr’s Resin (EMS) according to the manufacturer’s instructions. Sections approximately 1 μm thick were obtained with a Leica EM UC6 microtome (Leica) and then stained with 1% Toluidine Blue O before microscopic observation. To check the expression of fluorescent fusion proteins, plant materials mounted in water or transfected protoplasts were kept in WI solution [4mM MES (pH 5.7), 0.5M mannitol, and 20mM KCl] and were examined and photographed under a confocal microscope (Zeiss LSM710).

### Functional complementation of the yeast *lsg1* mutant

Full-length cDNA of *AtLSG1-2*, *AtLSG1-1*, and yeast LSG1 (*ScLSG1*), and truncated cDNA of *AtLSG1-2* were amplified by PCR and ligated into PRS415-GAD (Mumberg *et al.*, 1995). Plasmids containing intact or truncated AtLSG1-2, AtLSG1-1, wild-type yeast LSG1 (ScLSG1), or the empty vector (PRS415-GAD) were transformed into AJY1171 (MATalpha lsg1Δ::KanMX his3Δ1 leu2Δ0 ura3Δ0 lys2Δ0), which contained the plasmid pAJ626 (LSG1 URA3 CEN) (provided by Dr Arlen Johnson). Transformants were plated on Leu− medium, and colonies were diluted and dotted on Leu− medium containing 5-FOA and incubated for 5 d at 30 °C.

### Ribosome profiling analysis

Ribosome profiling analysis was performed with 10-d-old seedlings as previously described (Mustroph *et al.*, 2009).

### Isobaric tags for relative and absolute quantitation analysis

Roots of 10-d-old seedlings growing on MS agar media were used for isobaric tags for relative and absolute quantitation (iTRAQ) analysis. Protein sample preparation and analysis was performed as described in Zhao *et al.* (2014). The final proteomic data were derived from two biological replicates with three technical replicates each.

### Auxin treatment

Surface-sterilized seeds were directly plated on agar-medium plates with or without 1-N-naphthylphthalamic acid (NPA) supplement and grown for 10 d under the above-mentioned conditions. Phenotypes of the seedlings were observed and pictures were taken using a digital camera. GUS staining was performed as described above. For the auxin treatment, 5-d-old seedlings were transferred to auxin-containing medium. Length of primary roots was measured after 5 d of incubation in the growth chamber. For quantitative PCR analysis, 6-d-old seedlings were incubated in mock and 20 µM indole-3-acetic acid (IAA) for 2h.

### Accession numbers

Sequence data in this article can be found in the EMBL/Genbank data libraries under accession numbers Atlg08410 (*DIG6*/*AtLSG1-2*), At2g27200 (*AtLSG1-1*), At3g07050 (*NSN1*), and At1g52980 (*NUG2).*

## Results

### Isolation of the *dig6* mutant and map-based cloning of the *DIG6* gene

Because drought (water deficit) stress and phytohormone abscisic acid (ABA) can inhibit lateral root development in *Arabidopsis*, this trait was used to screen for mutants potentially involved in the drought stress response (Xiong *et al.*, 2006). One mutant, *dig6*, was isolated from an M_2_ population generated by EMS-mutagenized *Arabidopsis* in the Columbia *glabrous 1* (Col-*gl1*) background. This mutant is constitutively defective in lateral root growth regardless of the water potential or the ABA level in the growth media. Mutant seedlings grew slower than the wild-type and had a slightly yellowish colour (Supplementary Fig. S1A, B available at *JXB* online). In addition, mutants had fewer emerging lateral roots (LRs; Supplementary Fig. S1C) and displayed a strong incurvature phenotype in young leaves (Supplementary Fig. S1D).

**Fig. 1.**
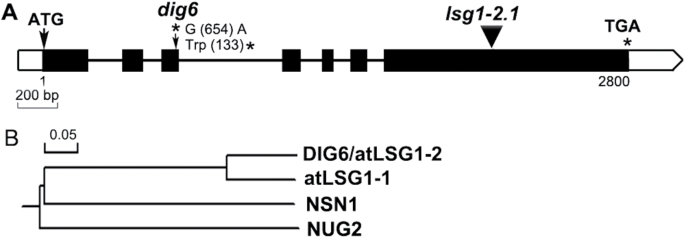
Structure of the *AtLSG1-2* gene and phylogenetic analysis of AtLSG1-2-related proteins in *Arabidopsis*. Genomic DNA structure of *AtLSG1-2* and the location of *AtLSG1-2* mutations. Exons, introns, and 5′or 3′UTRs are shown by black boxes, bold lines, and white boxes, respectively. Mutation sites are marked with arrows. (B) Phylogenetic tree of AtLSG1-2-related proteins generated by DNAman software.

To map the mutation, the mutant was crossed with Landsberg *erecta.* The mutation was initially mapped to a 2.754 Mbp region on Chromosome 1 between the simple sequence length polymorphic markers Chr1-1.070M and Chr1-3.824M (sequences of the markers are provided in Supplementary Table S1). Whole-genome DNA sequencing of the mutant was subsequently conducted. In the mapping interval, a G-to-A single nucleotide change was found in the At1g08410 gene (*AtLSG1-2*), which was predicted to generate a premature stop codon and would produce a truncated protein with 132 amino acids instead of the intact 589 amino acid length (Fig. 1A).

To confirm that the phenotypes observed in the mutant can be attributed to the loss of function of the *AtLSG1-2* gene, a T-DNA insertion allele (Salk_114083) in the Col-0 background was obtained. This mutant was described as *lsg1-2.1* (for simplicity also referred to as *lsg1-2* in this text hereafter) (Weis *et al.*, 2014). The F_1_ progeny plants of *dig6* and *lsg1-2* crosses showed the same phenotypes as *dig6* and *lsg1-2* (data not shown), indicating that AtLSG1-2 is responsible for the *dig6* mutant phenotypes.

Yeasts and humans have only a single copy of their respective AtLSG1 homologue, which is essential for cell viability (Hedges *et al.*, 2005; Reynaud *et al.*, 2005), while *Arabidopsis* has two homologues (Fig. 1B). *AtLSG1-1* (At2g27200) encodes a protein that shares 77.3% identity with AtLSG1-2 at the protein sequence level. A Blast search using AtLSG1-2 as a query sequence also found two other circularly permuted GTPases, NUCLEOSTEMIN-LIKE 1 (NSN1) and NUCLEAR/NUCLEOLAR GTPase 2 (NUG2), with only 21.1% and 29.8 % identity with AtLSG1-2, respectively. Nevertheless, the GTPase domain region of these proteins is better conserved relative to the rest of the protein (Supplementary Fig. S2). Previous studies showed that NSN1 is required for flower and leaf development (Wang *et al*., 2012a, 2012b) and NUG2 is involved in pre-60S ribosomal subunit maturation. The *AtNUG2* RNAi mutant has been shown to have increased sensitivity to cycloheximide treatment (Im *et al.*, 2011).

### Phenotypic characterization of the *lsg1-2* mutants

**Fig. 2.**
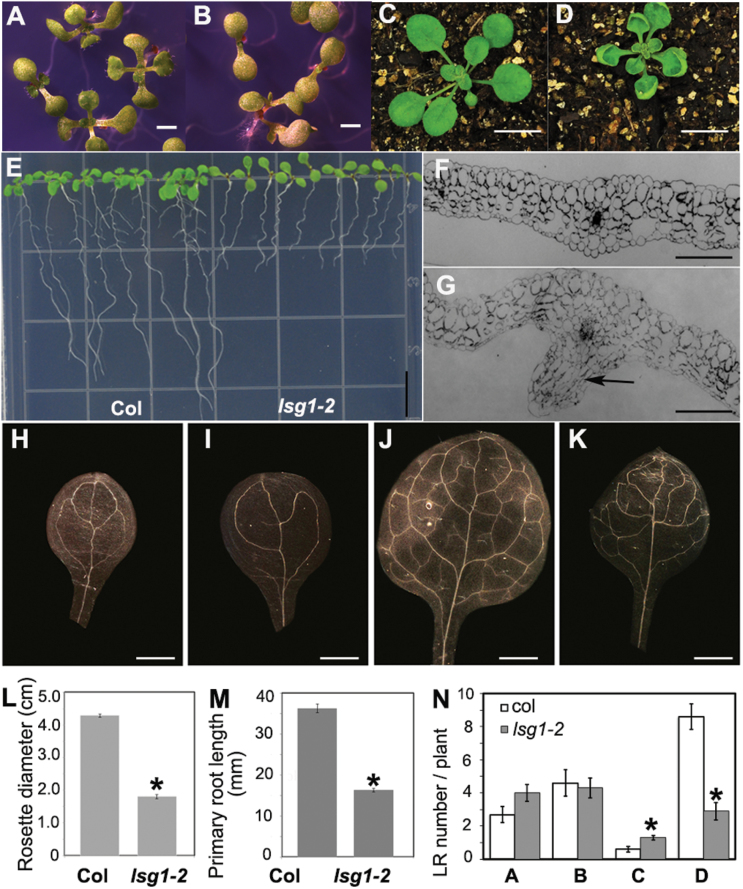
Phenotypes of the *lsg1-2* mutant. (A, B) Morphology of 12-d-old seedlings of Col-0 (A) and *lsg1-2* (B) growing on horizontally placed agar plates. Scale bars, 5mm. (C, D) Morphology of Col-0 (C) and *lsg1-2* (D) seedlings grown in soil. Scale bars, 1cm. (E) Phenotype of 12-d-old seedlings of Col-0 (A) and *lsg1-2* (B) growing on vertically placed agar plates. Scale bars, 1cm. (F, G) Transverse section of the fifth rosette leaf of Col-0 (F) and *lsg1-2* (G). Arrow indicates the region where extra cell division occurred. Scale bars, 200 µm. (H–K) Venations of cotyledons and the first pair rosette leaves in Col-0 (H, J) and *lsg1-2* (I, K). At least 10 plants were examined and the representative images are shown. Scale bars, 1mm. (L) The diameter of rosette leaves of 4-week-old plants growing in soil. (M) Primary root length of 12-d-old seedlings. (N) The number of LRs at different developmental stages (stages A–D) of 12-d-old seedlings. Data are means ± standard errors (*n*=8–16). *P<0.01, compared with wild-type plants (*t*-test).

Although *dig6* and *lsg1-2* mutants had identical morphological and developmental phenotypes (Fig. 2, Supplementary Fig. S1, see below), because of the possibility of interference from the *gl1* mutation on development and potential background mutations in the EMS-mutagenized *dig6* mutant, the *lsg1-2* allele was used for the characterization of mutant phenotypes.

Mutant leaves emerged later (Fig. 2A, B) and grew smaller (Fig. 2C, D) than wild-type leaves. Prior to bolting, *lsg1-2* mutants had significantly smaller stature than the wild-type (Fig. 2C, D, L), with markedly shorter primary roots (Fig. 2E, M, and Supplementary Fig. S1) and arrested lateral root growth (Fig. 2E and Supplementary Fig. S1). In 12-d-old seedlings, LRs were visible on wild-type plants but were barely visible in *lsg1-2* mutants. There are two possible explanations for decreased visible LRs in mutants: either LR growth is not being initiated or fewer LRs are elongated. By measuring the LR density, a higher density was found in the *lsg1-2* mutant (Supplementary Fig. S3), indicating that fewer visible LRs in the mutant cannot be explained by the failure of initiation. Therefore, LR number was examined at different developmental stages, for which a previous study was followed: LR development is divided into four stages (A to D) (De Smet *et al.*, 2003). In stage A, LRs are primordial with up to two cell layers; in stage B, LRs are between three cell layers and just prior to emergence; in stage C, LRs emerge but are shorter than 0.5mm; and in stage D, LRs are longer than 0.5mm (De Smet *et al.*, 2003). Among these stages of LRs, only stage D is visible to the naked eye. After examining LR number at all stages, significantly fewer LRs were found at stage D in the *lsg1-2* mutant (Fig. 2N). Thus, the reduced lateral root phenotype in the early developmental stage of the *lsg1-2* mutant was a result of a delay in LR emergence (Fig. 2E, 2N and Supplementary Fig. S1).

The most notable phenotype of *lsg1-2* mutants is the incurvature of rosette leaves (Fig. 2D and Supplementary Fig. S1D). Transverse thin sections were examined across the regions of fully expanded fifth leaves where the incurvature occurred and a significant increase in layers of spongy mesophyll cells was found in the abaxial side of mutant leaves (Fig. 2G), suggesting that extra cell division in the abaxial side of leaves is mainly responsible for the incurvature. Interestingly, the leaf incurvature phenotype was mainly exhibited at the early stages of plant development. When the inflorescence emerged, this incurvature phenotype could occasionally be seen in the newly emerged axillary leaves (Supplementary Fig. S1F). On the other hand, old rosette leaves and cauline leaves showed no sign of the incurvature phenotype. Noticeably, flatness of the cotyledon was not affected by *AtLSG1-2* deficiency (Fig. 2B).

Because vascular structure is closely related with leaf development, the structure of the wild-type and *lsg1-2* mutants was investigated. As shown in Fig. 2H–K, the vascular structure of cotyledons and the first pair of leaves in the *lsg1-2* mutant were very different from that of the wild-type plant. Vein loops were often open in the cotyledons of *lsg1-2* mutants, while those of the wild-type were typically closed (Fig. 2H, I). In the first pair of leaves, the distal part of the midvein was bifurcated in the *lsg1-2* mutant unlike the wild-type. Furthermore, the *lsg1-2* mutant had considerably fewer tertiary and quaternary veins compared with the wild-type (Fig. 2K).

### Expression patterns of *AtLSG1-2*

The expression of *AtLSG1-2* and *AtLSG1-1* was examined using qRT-PCR on RNA extracted from different organs of wild-type plants. *AtLSG1-2* was highly expressed in the flowers, moderately expressed in the roots, axillary leaves, rosette leaves, and siliques of young seedlings, and marginally expressed in the stalks of inflorescence and cauline leaves (Supplementary Fig. S4). *AtLSG1-1* was highly expressed in siliques, moderately expressed in roots, the flowers, and rosette leaves, and marginally expressed in the stalks of inflorescence, and cauline and axillary leaves of young seedlings (Supplementary Fig. S4). Generally, expression levels of *AtLSG1-2* were notably higher than those of *AtLSG1-1* in all the organs examined (Supplementary Fig. S4 available at *JXB* online).

**Fig. 3.**
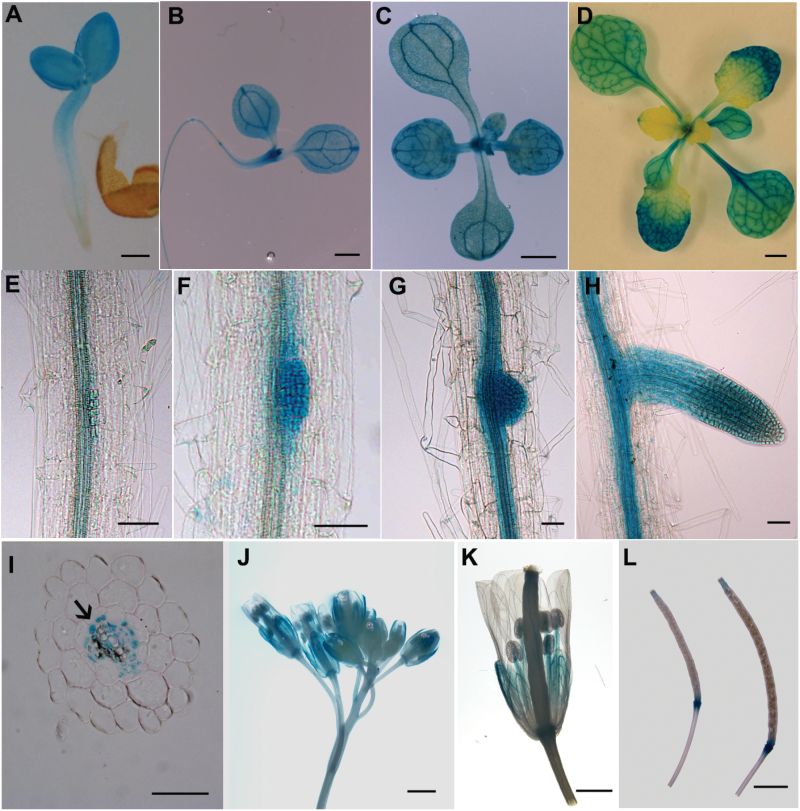
The expression pattern of *AtLSG1-2*. *pAtLSG1-2::GUS* expression was detected in 2- (A), 8- (B), and 12-d-old seedlings (C); 26-d-old soil-grown plant (D); LRs at stage A (E), C (F, G), and D (H); inflorescence (J, K); and siliques (L). (I) Transverse section of GUS-stained primary roots. Arrow indicates the xylem. Ten GUS-positive transgenic plants were checked and similar expression patterns were observed. A representative image is shown. Scale bars, 100 µm (A), 1mm (B–E), 50 µm (F–I), and 500 µm (J– L).

The expression pattern of *AtLSG1-2* during plant development was further studied by using the promoter-driven GUS reporter gene (Fig. 3A–L). Strong GUS signals in the entire cotyledon (Fig. 3A–D) and in emerging leaves (Fig. 3B) were observed. Strong signals were detected in all first pairs of leaves (Fig. 3A–D), but only in the distal part and with a concave pattern in second leaves (Fig. 3D). The GUS signal was highly expressed in the marginal region but minimally in the midvein region. This expression pattern was further demonstrated by qRT-PCR (Supplementary Fig. S5). In the primary root, *AtLSG1-2* was specifically expressed in vascular bundles along elongation and differentiation zones (Fig. 3A, E–I). Transverse section analysis showed that *AtLSG1-2* was mainly expressed in the stele but was excluded from the xylem (Fig. 3I). Because prominent LR phenotypes were observed in *lsg1-2* mutants, its expression was investigated in detail (Fig. 3E–H). It was found that *AtLSG1-2* is strongly expressed in lateral root cells of all stages examined (Fig. 3E–H) and in the inflorescence (Fig. 3I–J). In flowers, higher expression in filaments and sepals was observed (Fig. 3K). Strong signals were detected at the junction between the silique and the pedicel, while only low signals were detected at the tip of siliques (Fig. 3L). Despite the high expression level of *AtLSG1-2* in inflorescence or the silique, no visible phenotype was observed in these organs (Supplementary Fig. S6), probably because of the functional redundancy between *AtLSG1-2* and *AtLSG1-1*. Thus, it is evident that *AtLSG1-2* is highly expressed in newly emerged leaves, leaf veins, the vascular structure of the roots, and lateral root primordia where auxin also accumulates.

### AtLSG1-2 and AtLSG1-1 are localized in the cytosol in stably transformed plants

**Fig. 4.**
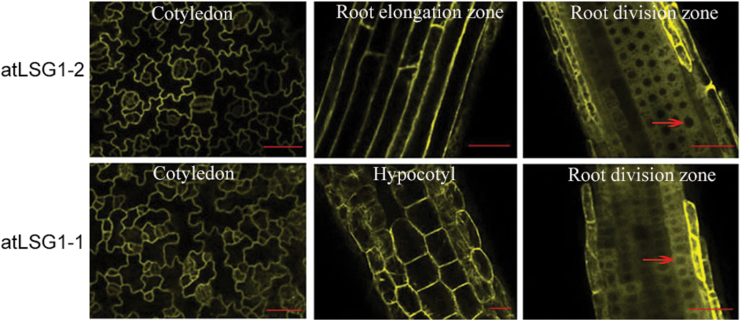
Subcellular localization of AtLSG1-2 and AtLSG1-1. The YFP signal was detected in the cytosol of cells in cotyledon, root division zone, root elongation zone, and hypocotyl of plants transformed with *35S::YFP-AtLSG1-1* or *35S::AtLSG1-2-YFP*. Arrows indicate nuclei. Scale bars, 50 µm. At least 10 transformed plants were examined.

Previous studies have shown that LSG1 proteins have different subcellular localization in yeast, human, and *Drosophila* cells (Gadal *et al.*, 2001; Reynaud *et al.*, 2005; Hartl *et al.*, 2013). To determine where they localize in plant cells, *35S::AtLSG1-2-YFP* and *35S::YFP-AtLSG1-2* constructs were created and transformed into the protoplasts of wild-type plants, and *dig6* and *lsg1-2* mutants. These constructs fully complemented the mutant phenotypes (Supplementary Fig. S7), suggesting that they were fully functional. In transiently expressed protoplasts, these proteins were localized in the nucleus and cytosol (data not shown), which is consistent with a recent study (Weis *et al.*, 2014). In stably transformed plants, the localization of fluorescence-tagged proteins was examined in different organs including the cotyledon, and the root elongation and division zones. Surprisingly, the localization of both AtLSG1-2 and AtLSG1-1 was limited to the cytosol in these organs (Fig. 4). This is particularly evident in the root division zone, where fluorescence signals were excluded from the hollow areas (i.e., nuclei) and were found in the cytoplasm (Fig. 4).

### Loss of function of *AtLSG1-2* affects ribosome biogenesis

**Fig. 5.**
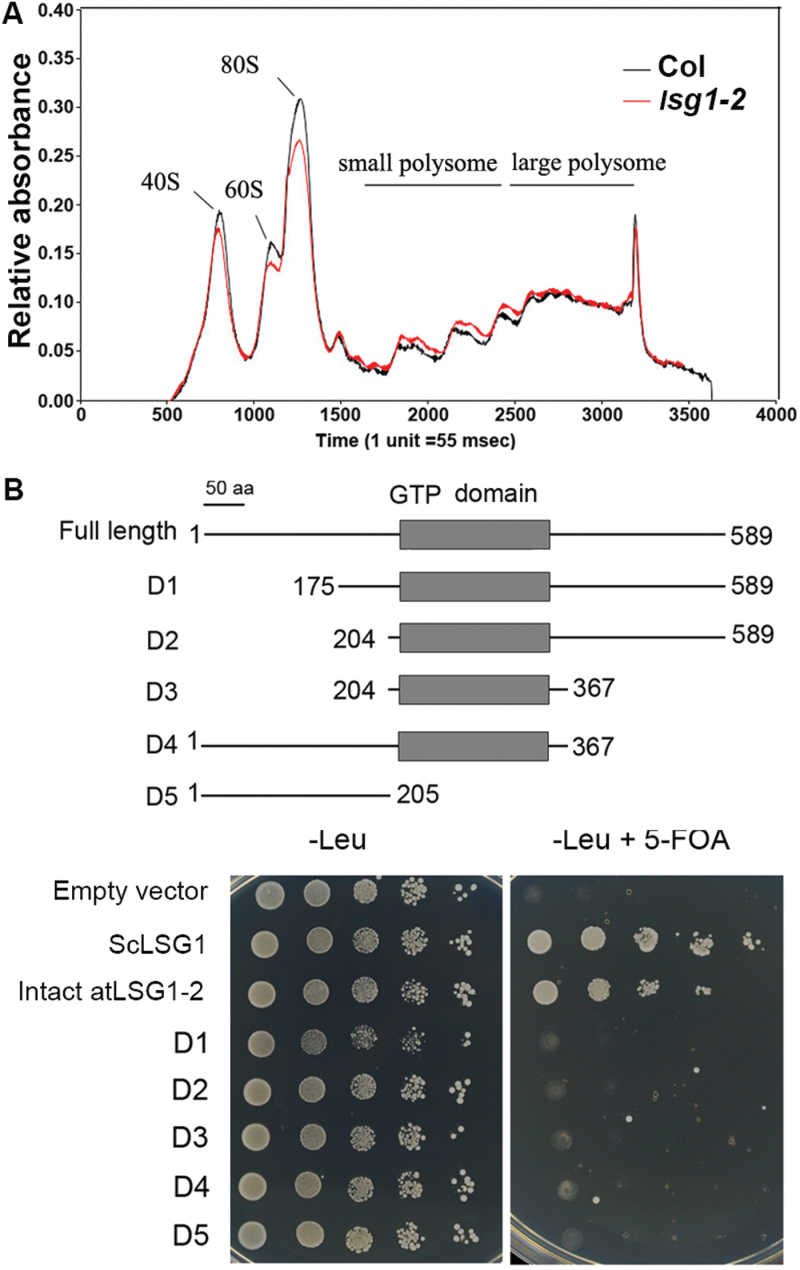
Ribosome profiling analysis and deletion analyses. (A) Ribosome profiling analysis using 10-d-old seedlings of Col-0 and the *lsg1-2* mutant. This experiment was repeated twice with similar results. (B) Deletion analysis. Various deletion fragments (D1 to D5, upper panel) of AtLSG1-2 used in yeast complementation assays are shown (lower panel). Empty vector (PRS415-GAD) and yeast LSG1 (ScLSG1) were used as negative and positive controls, respectively. At least three clones per construct were examined.

To investigate the impact of AtLSG1-2 deficiency on ribosome biogenesis in plants, ribosome levels were examined in 10-d-old mutant seedlings using ribosome profiling assays. The *lsg1-2* mutants were found to have decreased levels of 60S and 40S ribosomal subunits, and 80S monosomes, although polysome production was not affected (Fig. 5A). Thus, AtLSG1-2 is important for maintaining normal 60S and 40S ribosome biogenesis and 80S monosome assembly in plants.

The AtLSG1-2 protein includes an N- and a C-terminus and a GTP domain (Supplementary Fig. S2). In a previous study, certain regions were shown to be important for *LSG1* function in yeast; for example, the G1 motif is essential for GTP binding (Hedge *et al*., 2005), and a mutation in the predicted coil-coil region affects yeast growth (Hedge *et al*., 2005). To investigate which regions are important for AtLSG1-2 function, intact and a series of deletion constructs were made and these proteins were expressed in the yeast *lsg1* mutant. As shown in Fig. 5B, full-length cDNA from *AtLSG1-2* complemented the phenotype of the yeast *lsg1* mutant, similar to results from a recent study (Weis *et al.*, 2014). However, neither truncated protein rescued the yeast *lsg1* mutant, indicating that all the examined regions are essential for AtLSG1-2 function in ribosome biogenesis.

### Changes in the proteomic profile in the *lsg1-2* mutant

**Fig. 6.**
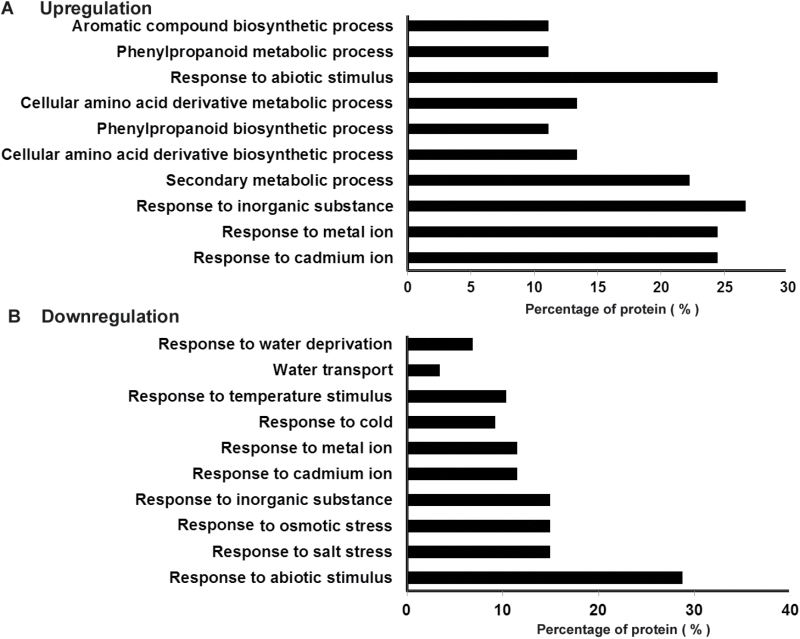
Functional classification of differentially expressed proteins in *lsg1-2* compared with wild-type Col-0. Proteins in the up-regulated (A) and down-regulated (B) groups were classified by DAVID software. The top 10 functional annotations ordered by the enrichment scores are presented. Two biological repeats and three technique repeats were performed for the iTRAQ analysis.

Because AtLSG1-2 is involved in ribosome biogenesis and *lsg1-2* mutants exhibited multiple developmental defects, it was anticipated that there would be changes in the proteome of the mutants. To probe changes in the proteomic profile of *lsg1-2* mutants, iTRAQ analysis was used to compare the global protein profile of 10-d-old-seedling roots of the wild-type and the *lsg1-2* mutant. Using a fold change greater than 1.3 and *P*<0.05 as the criteria for significance, 132 proteins in total were found to be differentially regulated (Supplementary Table S1 available at *JXB* online). Among them, 45 were significantly up-regulated and 87 were down-regulated in the mutant. A functional classification of differentially regulated proteins was made using the software DAVID (Huang *et al.*, 2009), and it was found that the up-regulated group was mainly associated with secondary metabolic process and in response to abiotic stimuli (Fig. 6A). The down-regulated group was enriched in proteins involved in response to abiotic stimuli and water transport (Fig. 6B). Interestingly, the iTRAQ analysis identified that 15 proteins involved in ribosome biogenesis were differentially regulated, which accounted for 11.3% of total differentially regulated proteins in the *lsg1-2* mutant. Among these, five have higher expression levels in the *lsg1-2* mutant (Table 1). These included two NOP56-LIKE pre-mRNA processing ribonucleoproteins (RNP), NUCLEOLIN LIKE 1 (ATNUC-L1), a 60S ribosomal protein, and NUCLEOPORIN 57 (NUP57). ATNUC-L1 is involved in rRNA processing, ribosome biosynthesis, and vascular pattern formation (Petricka and Nelson, 2007). All of the 10 down-regulated proteins are ribosomal proteins, including seven 60S and three 40S ribosomal proteins (Table 1). This is evidence that AtLSG1-2 loss of function affects the level of proteins involved in ribosome biogenesis. It was also noted that most of the down-regulated proteins involved in water transport are aquaporins. Among the 17 major aquaporin proteins in *Arabidopsis* (Péret *et al.*, 2012), seven were simultaneously repressed in the *lsg1-2* mutant. Notably, aquaporin proteins are regulated by auxin and are involved in lateral root emergence (Péret *et al.*, 2012).

**Table 1.** Differentially regulated proteins involved in ribosome biogenesis in the lsg1-2 mutant

Accession number	Protein	Fold change (*lsg1-2* vs Col)	*P* value
Up-regulated protein			
AT5G27120	NOP56-like pre-mRNA processing RNP	2.066	0.005
AT3G57150	Putative pseudouridine synthase (NAP57)	1.96	0.016
AT1G48920	Nucleolin like 1 (AtNUC-L1)	1.698	0.007
AT3G60245	60S ribosomal protein L37a-2	1.64	0.043
AT3G05060	NOP56-like pre-mRNA processing RNP	1.618	0.016
Down-regulated protein			
AT3G09200	60S acidic ribosomal protein P0-2	0.744	0.013
AT5G47930	40S ribosomal protein S27-3	0.727	0.022
AT5G64140	40S ribosomal protein S28-2	0.718	0.023
AT4G34670	40S ribosomal protein S3a-2	0.618	0.018
AT4G29410	60S ribosomal protein L28-2	0.616	0.009
AT2G44120	60S ribosomal protein L7-3	0.611	0.011
AT3G55280	60S ribosomal protein L23a-2	0.606	0.024
AT3G28900	60S ribosomal protein L34-3	0.395	0.006
AT1G61580	60S ribosomal protein L3-2	0.362	0.041
AT3G44590	60S acidic ribosomal protein P2-4	0.101	0.001

Values are means of three technical replicates expressed in relative fold changes.

### *AtLSG1-2* deficiency affects auxin distribution and response

**Fig. 7.**
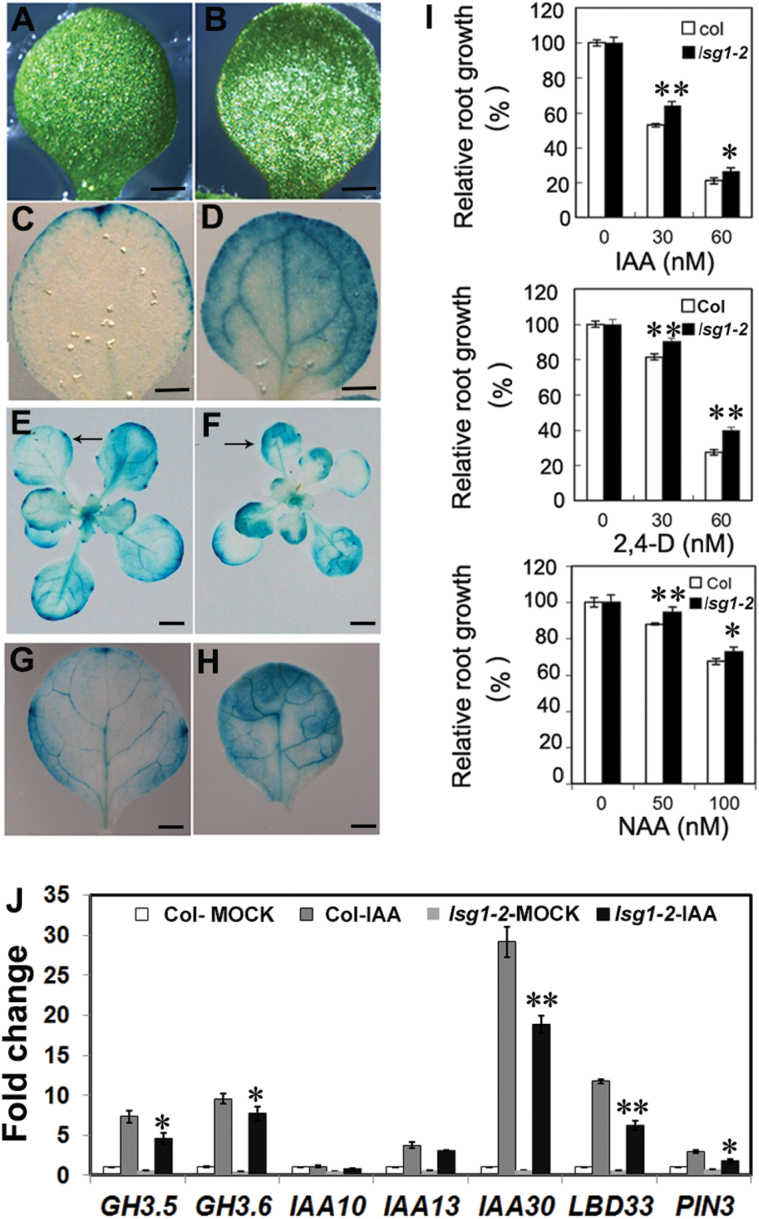
Auxin distribution in *lsg1-2* mutant leaves and auxin responses of the *lsg1-2* mutant. (A, B) Cotyledon phenotype of wild-type (Col-0) seedlings grown on MS medium without (A) or with (B) NPA. (C, D) *DR5::GUS* expression in the cotyledons of NPA untreated (C) and treated (D) wild-type seedlings. (E, F) *DR5::GUS* expression in the whole plant of Col-0 and *lsg1-2*. Arrows indicate first leaves. (G, H) Close-up view of *DR5::GUS* expression in the first leaf of Col-0 (g) and *lsg1-2* (h). (I) Auxin response of the *lsg1-2* mutant. Five-day-old seedlings grown on half-strength MS medium were transferred to media supplemented with the indicated concentrations of auxins (IAA, 2,4-D, and NAA) and grown for an additional 5 d. Primary root length was then measured and expressed relative to that under auxin-free conditions. Data are means ± standard errors (*n*=18). (J) Reduced levels of auxin-induced genes in the *lsg1-2* mutant as revealed by qRT-PCR analysis. *P<0.05, **P<0.01 (*t*-test) compared with wild-type plants. Scale bars, 1mm (A–D, G, H) and 2mm (E, F).

The *lsg1-2* mutants exhibited similar developmental defects as auxin-related mutants such as abnormal vein structure in leaves and cotyledons, short primary roots, and delayed LR emergence. The DR5::GUS reporter was employed to monitor the distribution of auxin in *lsg1-2* mutant leaves. The reporter gene was introduced into *lsg1-2* by genetic crossing and its expression was examined. The GUS staining pattern in the mutant was distinctly different from that in the wild-type. High expression of the *DR5::GUS* reporter gene was observed in the leaf border of *lsg1-2* mutant leaves (Fig. 7F, H), similar to NPA-treated seedlings (Fig. 7C, D). The accumulation of auxin or an increased auxin response may have triggered the extra cell division activity that was observed in the abaxial side of *lsg1-2* mutant leaves, causing the leaf incurvature phenotype in *lsg1-2* mutants.

The sensitivity of *lsg1-2* mutants to auxin treatments was investigated. Primary root growth of the wild-type and the *lsg1-2* mutant was examined with seedlings growing on MS medium containing either IAA, 2,4-Dichlorophenoxyacetic acid (2,4-D), or 1-naphthaleneacetic acid (NAA). Auxin influx carriers mediate the influx of exogenous IAA and 2,4-D into the roots, whereas NAA can freely penetrate into the cells in a carrier-independent manner (Estelle, 1998). As shown in Fig. 7I, the *lsg1-2* mutant consistently exhibited reduced sensitivity to these auxins.

Next, the response of *AtLSG1-2* to auxin was examined in terms of auxin-inducible gene expression. Six-day-old wild-type and *lsg1-2* seedlings were treated with 20 μM IAA for 2h and the expression levels of several auxin-responsive genes were analysed using qRT-PCR. Although the expression levels of most genes were induced to various extents by auxin, they were induced to a lesser extent in the *lsg1-2* mutant than in the wild-type (Fig. 7J).

### The *lsg1-2* mutant had reduced levels of auxin influx and efflux carriers, and impaired auxin transport

**Fig. 8.**
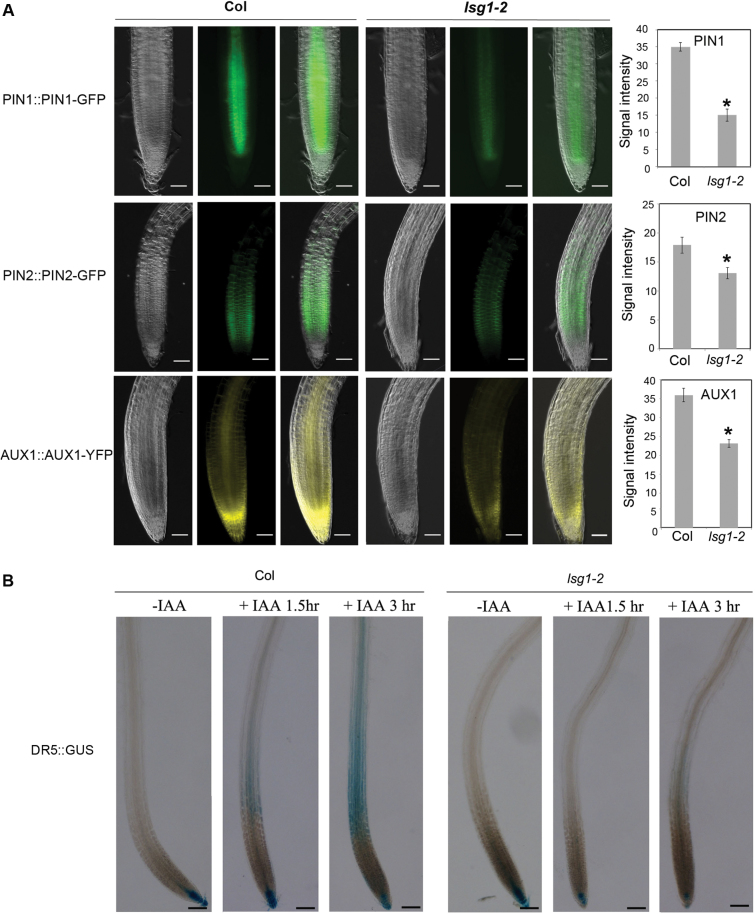
The *lsg1-2* mutant has reduced levels of auxin transporters and displays auxin transport defects. (A) Expression of *pPIN1::PIN1-GFP*, *pPIN2::PIN2-GFP*, and *pAUX1::AUX1-YFP* in roots of Col-0 and *lsg1-2*, and quantification of the fluorescence intensity. For each genotype- reporter, the left panel presents a bright-field image of the root; the middle panel presents the GFP or YFP image; and the right panel shows the merged image of bright-field and GFP/YFP images. Scale bars, 50 µM. Fluorescence intensities were quantified using Image J. Data represent means ± standard error (*n* = 10). *P<0.01 (*t*-test) compared with wild-type plants. (B) The auxin transport process was monitored using *DR5::GUS* reporter in wild-type (Col-0) and *lsg1-2* mutant roots. Root tips were treated with IAA for 1.5 or 3h. Roots were then stained for reporter gene expression. Scale bars, 100 µM. At least 10 plants were examined with similar results. Representative images are shown.

The movement of auxin within plants is mediated by a group of transporters. Thus, it was examined whether the expression of auxin transporters was affected in the *lsg1-2* mutant. The GFP-tagged auxin efflux carriers *pPIN1::PIN1-GFP* and *pPIN2::PIN2-GFP*, and the auxin influx carrier *pAUX1::AUX1-GFP* were introduced into the *lsg1-2* mutant by genetic crossing. It was found that the expression levels of PIN1, PIN2, and AUX1 were significantly lower in *lsg1-2* mutants compared with those in the wild-type (Fig. 8A). Using *DR5::GUS* as a reporter, auxin transport between the wild-type and mutants was compared. After root tips were treated with IAA for 1.5h, *DR5::GUS* signals became easily visible in the upper part of the wild-type but not in mutant roots (Fig. 8B). After an additional 1.5h, more signals were detected in the upper part of the wild-type root but only faint signals were detected in mutant roots (Fig. 8B). These data indicated that auxin transport was also impaired in the mutants.

### Loss of *AtLSG1-2* activates compensatory expression of *AtLSG1-1*

**Fig. 9.**
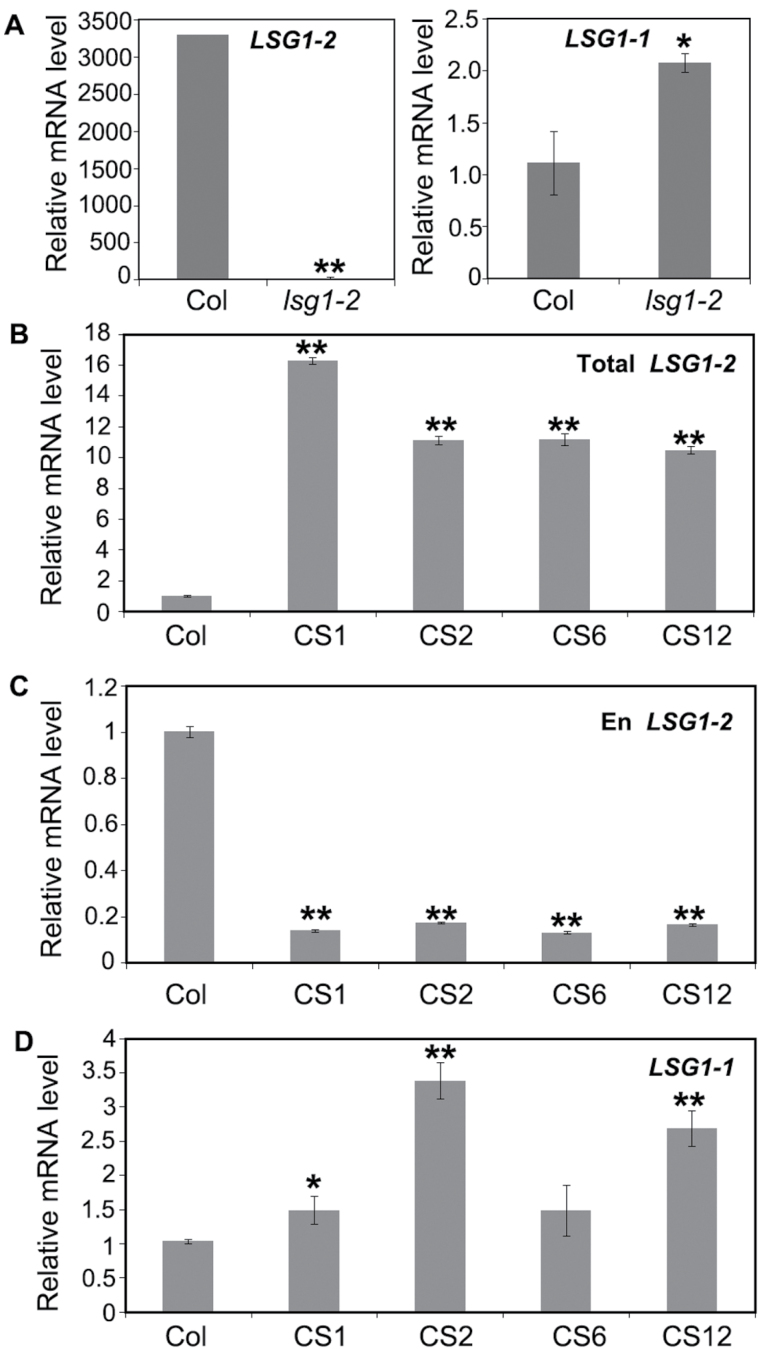
Expression levels of *atLSG1-2* and *atLSG1-1* in the wild-type, *lsg1-2*, and cosuppression plants. (A) Relative expression levels of *atLSG1-2* and *atLSG1-1* in wild-type and *lsg1-2* plants. (B, C) Relative expression level of *atLSG1-2* from both the endogenous gene and the transgene (B) and from the endogenous gene only (C). (D) Relative expression level of *atLSG1-1* in (B–D), Col, wild-type Col-0; CS1, CS2, CS6, and CS12 independent transgenic plants expressing *35S::atLSG1-2-YFP* that showed cosuppression phenotypes. Gene expression level was measured by qRT-PCR and expressed relative to that of the *UBQ10* gene. Data are means ± standard error from three biological replicates. *P<0.05, **P<0.01 (*t*-test) compared with the wild-type plants.

AtLSG1-2 and atLSG1-1 have a high degree of amino acid identity and also share overlapping gene expression patterns. The relatively mild phenotypes of *lsg1-2* mutants suggest that AtLSG1-1 might partly compensate for the loss of AtLSG1-2. The expression level of *AtLSG1-1* was examined in the leaves of *AtLSG1-2* knockout line by qRT-PCR and was found markedly increased in *lsg1-2* (Fig. 9A). Next, the expression levels of *AtLSG1-1* in *AtLSG1-2* cosuppression lines that were transformed with the *AtLSG1-2* overexpression construct were investigated yet showed phenotypes similar to those of *lsg1-2* mutants, likely due to silencing of the endogenous gene. In these plants, the total expression level of *AtLSG1-2* from both the endogenous gene and the transgene was significantly higher (Fig. 9B), but the expression level of endogenous *AtLSG1-2* was greatly decreased (Fig. 9C). In contrast, the expression level of *AtLSG1-1* in these plants increased (Fig. 9D), consistent with the notion that *AtLSG1-2* loss of function could induce increased expression of *AtLSG1-1*.

## Discussion

In this study, a genetic screen and position cloning identified *AtLSG1-2* as an important protein involved in several developmental processes in *Arabidopsis*. Both here and in a previous study, AtLSG1-2 is shown to share similar conserved functions in ribosome biogenesis as its homologues in other organisms. However, this study showed that AtLSG1 proteins also have some distinct functions. The ribosome profiling experiments revealed that the *lsg1-2* knockout mutant had lower levels of 40S and 60S ribosome subunits, and the 80S monosome, while the polysome level was not affected (Fig. 5A), which is different from yeast *lsg1* dominant-negative mutants and temperature-sensitive mutants where polysomes were also affected (Kallstrom *et al.*, 2003; Hedge *et al*., 2005). This might be explained by differences between species or the presence of *AtLSG1-1* in *Arabidopsis*. In the *lsg1-2* single mutant, some phenotypes are similar to the *Drosophila ns3* weak allele mutant, such as growth arrest and small stature, although these two proteins localize in different compartments. This phenotype similarity is probably related to defective ribosome biogenesis. Interestingly, the growth defects of the *ns3* mutant can be rescued by the overexpression of AKT1 (Kaplan *et al.*, 2008), a kinase that is the central effector of the insulin pathway. It is still unclear if the insulin pathway exists in plants, and no AKT1 homologue has been found in *Arabidopsis*. Thus, *AtLSG1-2* probably uses a different pathway to control body size in *Arabidopsis*.

This study showed that *lsg1-2* mutants exhibited some auxin-related phenotypes including stunted root growth, delayed LR emergence, and altered auxin response and transport. These phenotypes imply that certain auxin-related or auxin-regulated proteins might be differentially regulated. Nonetheless, auxin-related proteins were not detected in the proteomics data, perhaps because the expression level of these proteins was too low to be detected under these experimental conditions, where likely only abundant proteins could be detected. Interestingly, many aquaporin proteins, which are negatively regulated by auxin (Péret *et al.*, 2012), were significantly down-regulated in the *lsg1-2* mutant. Because LR emergence depends on the spatial and temporal distribution of aquaporins, knocking out or overexpressing the aquaporin gene *PIP2;1* causes delayed LR emergence (Péret *et al.*, 2012). This particular gene was found to be down-regulated in the *lsg1-2* mutant. Thus, altering the expression of these aquaporins may contribute to the delayed LR emergence observed in *lsg1-2* mutants.

Previous studies have shown that several ribosome mutants display auxin-related phenotypes (Nishimura *et al.*, 2005; Rosado *et al.*, 2012). For example, loss of function of the ribosome protein RPL24 causes defects in gynoecium development, similar to the auxin response factor mutants *ettin* and *monopteros* (Nishimura *et al.*, 2005). The *rpl4d* and *rpl5a* mutants showed altered vascular structure in leaves and stunted primary root growth (Rosado *et al.*, 2012), phenotypes that have been associated with the translational control of auxin response factors by their upstream ORFs (uORFs) (Nishimura *et al.*, 2005; Rosado *et al.*, 2012). This study demonstrated the roles of AtLSG1-2 in ribosome biogenesis and found that the level of 10 ribosomal proteins was simultaneously decreased in the *atlsg1-2* mutant. Collectively, these defects may compromise the ability of ribosomes to translate certain uORF-containing regulatory proteins. It is possible that the regulation of the auxin response by AtLSG1-2 is also through similar mechanisms by affecting the translation of uORF-containing regulatory genes.

A recent study showed that AtLSG1-2 is localized in the nucleus and cytosol in transiently transformed protoplasts (Weis *et al.*, 2014). Here, its localization pattern was examined in stably transformed plants and it was found that, similar to yeast LSG1, both AtLSG1-2 and AtLSG1-1 are restricted to the cytoplasm (Fig. 4). Because the constructs used here complemented the *lsg1-2* mutant phenotypes, the GFP-fused AtLSG1 protein is functional. The different localization patterns found in protoplasts transiently expressing the AtLSG1 proteins and transgenic plants stably expressing these proteins likely result from different expression levels of their fusion proteins. It is known that transient protoplast transformation requires a high concentration of plasmid DNA, thus the fluorescence fusion proteins are probably excessively expressed in these cells. Alternatively, differences in cellular state between isolated protoplast cells and intact cells from living plants could play a role. To more accurately identify the subcellular localization of AtLSG1 proteins in plants, one may need to use their native promoter-driven fluorescent proteins expressed in stably transformed plants or use subcellular fractionation approaches in the future.

In yeast, *Drosophila*, and humans, LSG1 deficiency is typically lethal. However, in *Arabidopsis* there are two *LSG1* genes, and the loss of either *AtLSG1* gene does not cause death, indicating that the two *LSG1* genes have redundant functions. Although *AtLSG1-1* showed low expression levels and *atlsg1-1* displayed subtle phenotypes (Weis *et al.*, 2014), increased levels of *AtLSG1-1* were observed in the *lsg1-2* knockout or knockdown mutants, suggesting there may exist a compensatory mechanism in these mutants. In fact, two compensatory mechanisms have been found in other organisms (Gu *et al.*, 2003; Hanada *et al.*, 2011). The first mechanism is associated with duplicate genes because sequence similarity is positively correlated with compensation frequency (Gu *et al.*, 2003); and the second mechanism is associated with alternative pathways or regulatory networks. This investigation shows that *AtLSG1* follows the first type of mechanism because of the high similarity of the two protein sequences. However, compensation does not completely fulfil the requirement for LSG1 in the *lsg1-2* mutant because the mutant still showed mild phenotypes. The compensatory mechanism has also been experimentally demonstrated in other mutants; for example, in the ribosome protein mutant *rpl4a*, its paralogue RPL4D protein levels were found to accumulate and vice versa (Rosada *et al*., 2012), and the knockout of *Rpl22* resulted in the upregulation of *Rpl22-like1*, the paralogue of *Rpl22* in mice (O’Leary *et al.*, 2013). This compensatory mechanism may contribute to the plasticity of adaptation of organisms.

## Supplementary data

Supplementary data are available at *JXB* online.

Fig. S1. The phenotypes of the *dig6* mutant.

Fig. S2. Multiple sequence alignment of AtLSG1-2-related proteins in *Arabidopsis*.

Fig. S3. Lateral root density.

Fig. S4. Expression of *AtLSG1-2* and *AtLSG1-1* in different organs examined by qRT-PCR.

Fig. S5. Expression of *AtLSG1-2* in leaf middle and margin regions.

Fig. S6. The *lsg1-2* mutant displayed no visible phenotype at late development stages.

Fig. S7. *35S::YFP-AtLSG1-2* complements the phenotypes of the *lsg1-2* mutant.

Table S1. Differentially regulated proteins identified by iTRAQ analysis.

Table S2. List of primers used in this study.

Supplementary Data
