# Supplementary material for: The Arabidopsis gene DIG6 encodes a large 60S subunit nuclear export GTPase 1 that is involved in ribosome biogenesis and affects multiple auxin-regulated development processes
Source: J Exp Bot. 2015 Aug 13;66(21):6863–75. doi: 10.1093/jxb/erv391 (PMC4623693; doi:10.1093/jxb/erv391)
Supplement: Supplementary Data [file supp_66_21_6863__index.html]

The Arabidopsis gene DIG6 encodes a large 60S subunit nuclear export GTPase 1 that is involved in ribosome biogenesis and affects multiple auxin-regulated development processes — The Arabidopsis gene DIG6 encodes a large 60S subunit nuclear export GTPase 1 that is involved in ribosome biogenesis and affects multiple auxin-regulated development processes — Supplementary Data 

# The *Arabidopsis* gene *DIG6* encodes a large 60S subunit nuclear export GTPase 1 that is involved in ribosome biogenesis and affects multiple auxin-regulated development processes

## Supplementary Data

Data files

- Supplementary Data - Supplementary Data
- Supplementary Data - Supplementary Data
