# Supplementary material for: The Arabidopsis gene DIG6 encodes a large 60S subunit nuclear export GTPase 1 that is involved in ribosome biogenesis and affects multiple auxin-regulated development processes
Source: J Exp Bot. 2015 Aug 13;66(21):6863–75. doi: 10.1093/jxb/erv391 (PMC4623693; doi:10.1093/jxb/erv391)
Supplement: Supplementary Data [file supp_erv391_jexbot148007_file001.pdf]

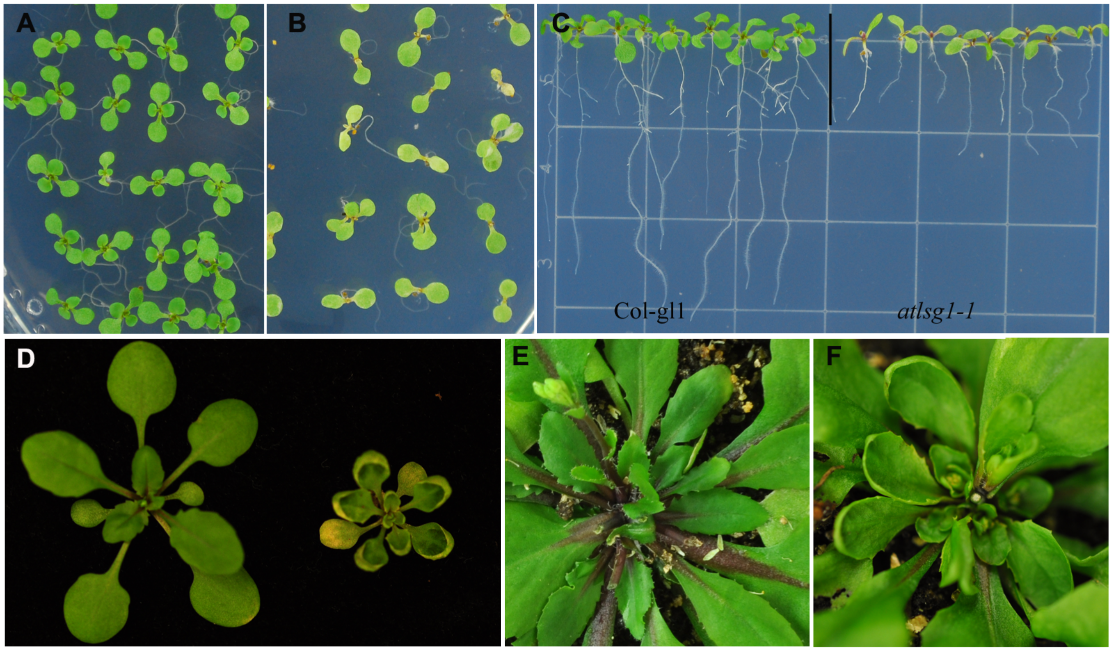

Figure S1. The phenotypes of *dig6* mutant. (A-C) Twelve-day-old seedlings grown on horizontally (A-B) or vertically (C) placed agar plates. Seedlings in (A) and on the left of panel (C) are wild type (Col-*gl1*) and in (B) and on the right of panel (C) are *dig6*. (D) Twenty-eight-day-old Col-*gl1* (left) and *dig6* (right). (E-F) Newly emerged axillary leaves of Col-*gl1* (E) and *dig6*.

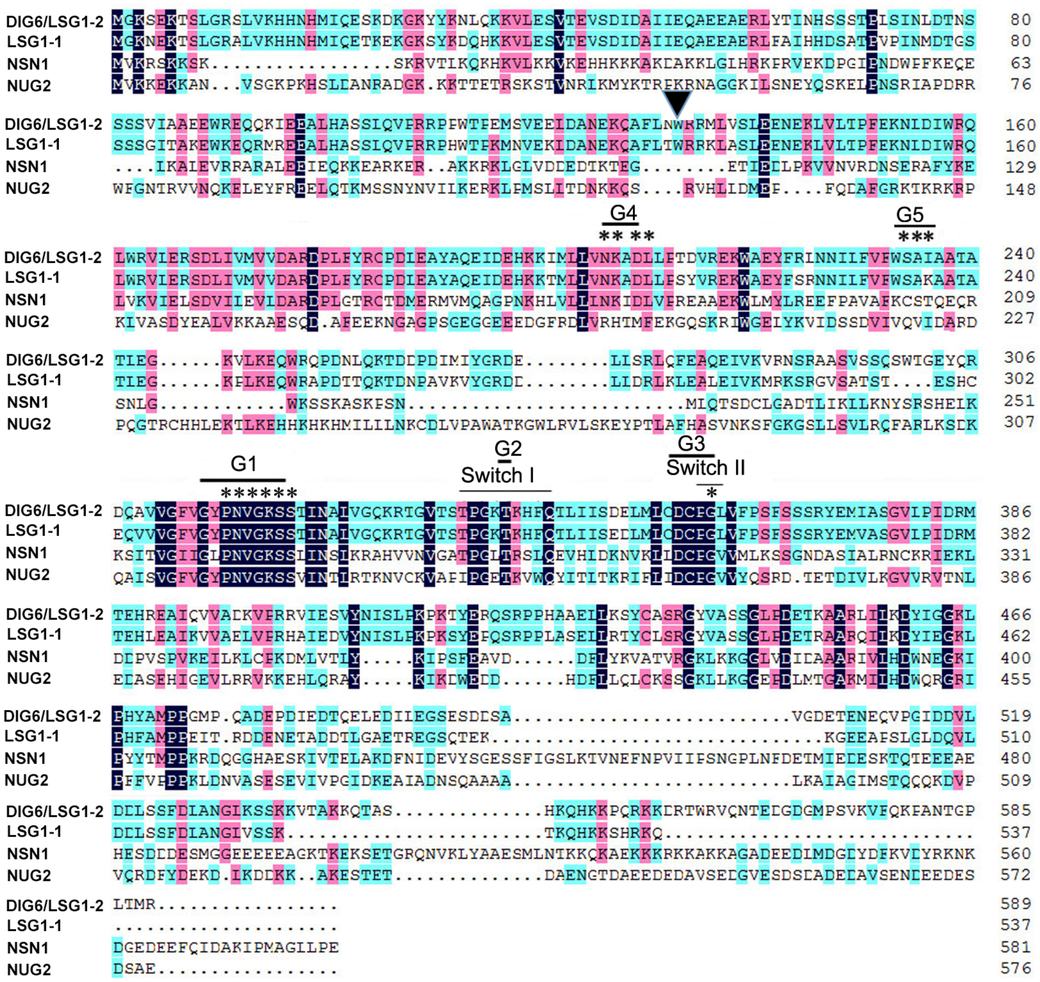

Figure S2. Multiple alignment of AtLSG1-related proteins. The accession numbers of DIG6/LSG1-2, LSG1-1, NSN1 and NUG2 are Atlg08410, At2g27200, At3g07050, and At1g52980, respectively. The putative motifs were marked by black lines above the sequence. Asterisks represent GTP/Mg^{2+} binding sites.

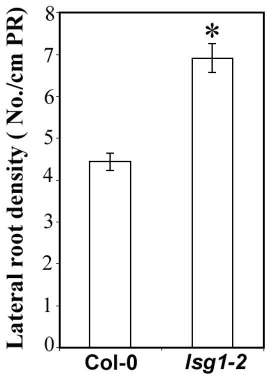

Figure S3. Lateral root density. Lateral root number was counted in the primary root of 12-day-old seedlings. Asterisk indicates significant difference from wild type plants (*t*-test, * p<0.01).

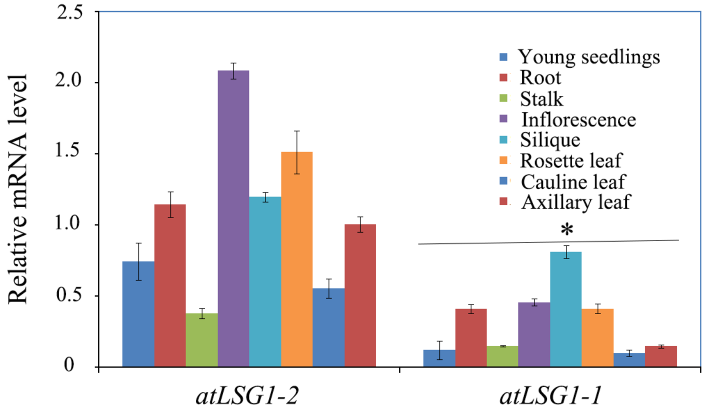

Figure S4. The tissue-specific expression pattern of *atLSG1-1* and *atLSG1-2*. RNA was extracted from 12-day-old seedlings and different organs of mature plants. Real time RT-PCR was carried out. Student t-test was carried out for comparing the different expression levels of *atLSG1-1* and *atLSG1-2* in various organs. Asterisk indicates significant differences between two genes (*t*-test, * p<0.01).

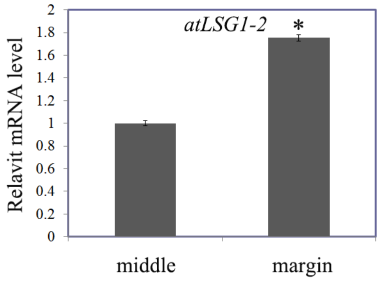

Figure S5. The expression of *atLSG1-2* in leaf middle and margin regions. Total RNA was extracted from the leaf mid vein or margin regions, and real time RT-PCR was carried out using *atLSG1-2* specific primers. Asterisk indicates significant difference from wild type plants (*t*-test, * $p<0.01$).

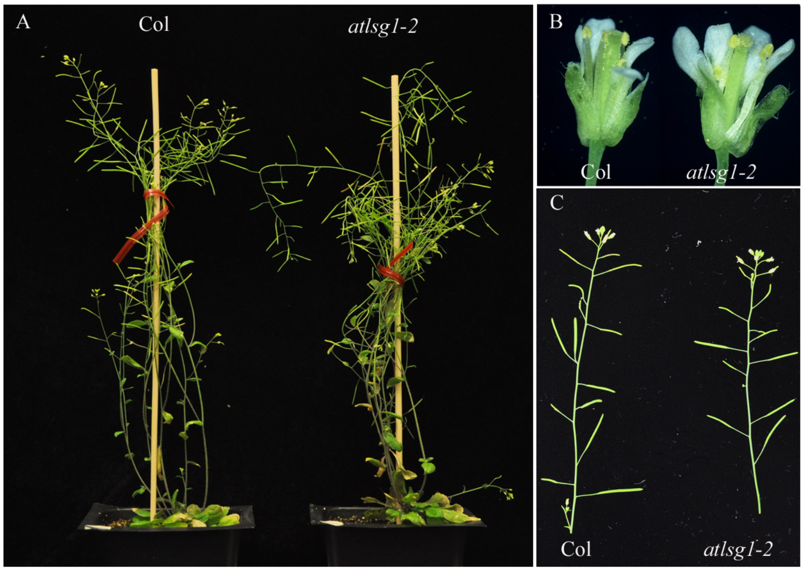

Figure S6. The *atlsg1-2* mutant displayed no visible phenotype at late development stages. Morphology of (A) the mature plants, (B) flowers, and (C) siliques of the wild type Col-0 and *atlsg1-2*.

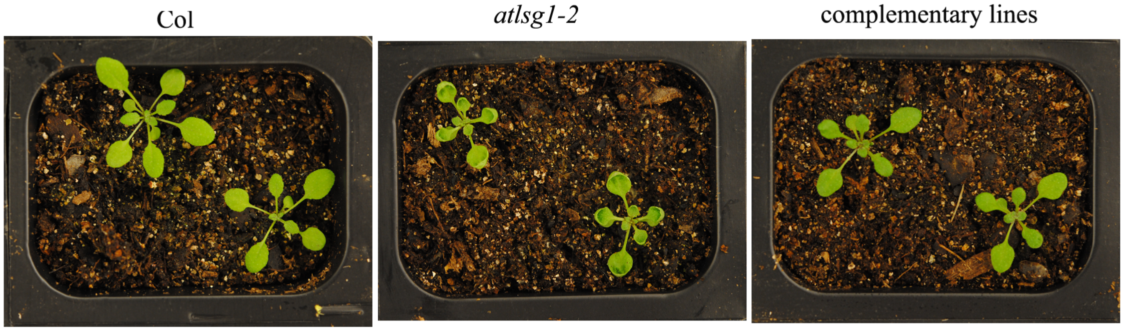

Figure S7. *35S::YFP-AtLSG1* complements the phenotypes of the *lsg1-2* mutant.

Table S2 Primer list in this study

purpose	gene name	sequence
for mapping	Chr1-1.070MF	GAGCTGGTGAGAGCCAAAGTTTCC
	Chr1-1.070MR	CACGTAGAAGCGATCACAGAGATAC
	Chr1-3.824MF	CCCTAGTCGAGTATTTACAGCGACTC
	Chr1-3.824MR	TACAGCCATTAGGTCTTATAACCGAT
for checking the transcripts of	OXH89-F	CTCGAGAACTACTGCGACAGG
	OXH76-R	TGGAGACAACCCGATAACTTG
for vector construction	AtLSG1-F	CACCATGGGGAAGAGCGAAAAGAC
	AtLSG1-R	CCGCATCGTAAGAGGACCTG TG
	AtLSG1-R	TCACCGCATCGTAAGAGGAC
	AtLSG1 like-F	CACCATGGGGAAGA ACGAGAAAAC
	AtLSG1 like-R	TTGTTTCCTATGTGACTTCTT
for making yeast constructs	LSG1-F	CTGACTAGTAAAATGCCACCAAAAGAAGCTCC
	LSG1-R	TCTGTCGACCTAATTATTTTCAATGCTAAAAACT
	AtLSG1-F	CTGACTAGTAAAATGGGGAAGAGCGAAAAGAC
	AtLSG1-R	ACTGTCGACGGATCCTCACCGCATCGTAAGAGGAC
	AtLSG1 like-F	CTGACTAGTAAAATGGGGAAGAACGAGAAAAC
	AtLSG1 like-R	ACTGTCGACGGATCCTCATTGTTTCCTATGTGACTT
for making PAtLSG1::GUS construct		CACCTTGCAGACTGTGAGTTGGAT
		CTCTCTGTTC TCAAGTAAACC
For making artificial microRNA	I miR-s	gaTTACTTGAAGACTACTCGCGTtctctcttttgtattcc
	II miR-a	gaACGCGAGTAGTCTTCAAGTAAtcaaagagaatcaatga
	III miR*s	gaACACGAGTAGTCTACAAGTATtcacaggtcgtgatatg
	IV miR*a	gaATACTTGTAGACTACTCGTGTtctacatatatattcct
For real-time PCR	PIN3-F	CGCCGAGAATGGTCTAAACAAA
	PIN3-R	AAAGCGACGAGAGCCCAAATAA
	IAA10-F	GTGAAGGTGACAATGGACGG
	IAA10-R	GGAACATCTCCTACAAGCATCC
	IAA20-F	GAGGAGGAAGAAGAGAATGAGTGT
	IAA20-R	TCAGCCCAGAGAATGGATGC
	IAA30-F	GAACCTAAGCACGGACCTGA
	IAA30-R	TCATCATTTCCTCTGCCGCA
	IAA13-F	GATGCTTGTTGGTGATGTTCCA
	IAA13-R	TGCTTTCGCTGTCTCTCGTT
	GH3.6-F	ATGTCCTAAGCGTGGCTGGT
	GH3.6-R	GGCGTGTTACCGTTCAAGCA
	LBD33-F	GATTGAGTTTCTCGGGTCACAG
	LBD33-R	CCCATCATAGCAGTTTCTTCCG
	ACS6 -F	TCCAGGCGGTTCGTTCCATT
	ACS6 -R	AGCCATCGGTTTAGTCTCCTCC
